# Supplementary material for: Bioactive Clerodane Diterpenoids from the Leaves of Casearia coriacea Vent
Source: Molecules. 2023 Jan 25;28(3):1197. doi: 10.3390/molecules28031197 (PMC9918898; doi:10.3390/molecules28031197)
Supplement: Supplementary file 1 [file molecules-28-01197-s001.zip › molecules-2134837-supplementary.docx]

Supporting informations

**Bioactive clerodane Diterpenoids from the leaves of *Casearia coriacea* Vent.**

Allison Ledoux^1^, Carla Hamann^1-2^, Olivier Bonnet^1^, Kateline Jullien^1^, Joëlle Quetin-Leclercq^3^, Alembert Tchinda^1^, Jacqueline Smadja^4^, Anne Gauvin-Bialecki^4^, Erik Maquoi^2^, Michel Frédérich^1^

^1^Laboratory of Pharmacognosy, Center of Interdisciplinary Research on Medicines, CIRM, University of Liège, Avenue Hippocrate 15, 4000 Liège, Belgium

^2^Laboratory of Biology of Tumor and Development, GIGA/CIRM, University of Liège, Avenue Hippocrate 15, 4000 Liège, Belgium

^3^Pharmacognosy Research Group, Louvain Drug Research Institute, LDRI, Université catholique de Louvain, UClouvain, Avenue E. Mounier, B1 72.03, B-1200, Belgium

^4^Laboratoire de Chimie des Substances Naturelles et des Sciences des Aliments, Université de Réunion, Avenue René Cassin 15, BP 7151, 97715 Saint-Denis, La Réunion, France

**Correspondence**

Dr. Ledoux Allison, Laboratory of Pharmacognosy, Center of Interdisciplinary Research on Medicines, CIRM, University of Liège, Avenue Hippocrate 15, 4000 Liège, Belgium. E-mail: [Allison.ledoux@uliege.be](mailto:Allison.ledoux@uliege.be) Phone : +32 4366 43 90

**ORCID**

Ledoux Allison: 0000-0002-4052-6336

Table S1 : ^13^C-NMR data information of compounds **1**-**3** and their comparison with literature.

| Carbone NMR data | Caseamembrin T | Compound 1 | Corymbulosine I | Compound 2 | Caseamembrin E | Isocaseamembrin E | Compound 3 |
| --- | --- | --- | --- | --- | --- | --- | --- |
| 1 | 26,9 | 27,8 | 26,8 | 28 | 26,3 | 26,8 | 28 |
| **2** | **66,2** | **66,4** | **66,1** | **66,6** | **70,5** | **66,3** | **66,6** |
| 3 | 121,8 | 121,9 | 121,9 | 121,9 | 124,3 | 121,9 | 121,9 |
| 4 | 145,1 | 145,8 | 145,4 | 145,8 | 144,4 | 145,5 | 145,8 |
| 5 | 53,7 | 53,8 | 53,8 | 54,4 | 53,8 | 53,9 | 54,4 |
| 6 | 73,1 | 72,9 | 73 | 73 | 74,3 | 73,1 | 73 |
| 7 | 36,5 | 37,4 | 37,2 | 37,9 | 37,4 | 37,3 | 37,4 |
| 8 | 37,1 | 37,5 | 37,5 | 37,5 | 37,6 | 37,6 | 37,6 |
| 9 | 36,5 | 38,2 | 37,3 | 36,4 | 38,2 | 37,5 | 37,4 |
| 10 | 37,4 | 36,6 | 36,5 | 36,5 | 41,2 | 36,5 | 36 |
| 11 | 28,1 | 26,8 | 28 | 26,8 | 27,7 | 28 | 26,7 |
| 12 | 23,8 | 23,9 | 23,7 | 23,5 | 23,8 | 23,8 | 23,5 |
| 13 | 145,3 | 145,2 | 145,1 | 146 | 145,2 | 145,1 | 146 |
| 14 | 140,4 | 140,8 | 140,4 | 140,8 | 140,3 | 140,5 | 140,8 |
| 15 | 112,3 | 112,6 | 112,2 | 115,8 | 112,6 | 112,3 | 112,6 |
| 16 | 115,4 | 115,8 | 115,5 | 112,6 | 115,4 | 115,6 | 115,8 |
| 17 | **25,3*** | 15,7 | 15,7 | 15,9 | 15,7 | 15,8 | 15,5 |
| 18 | 95,6 | 95,7 | 95,5 | 95,8 | 95,1 | 95,6 | 95,5 |
| 19 | 97,9 | 97,8 | 97,8 | 97,8 | 97,6 | 97,9 | 97,8 |
| 20 | **15,7*** | 25,3 | 25,5 | 25,7 | 25,5 | 25,5 | 25,6 |
| 1' | 173,1 | 173,3 | 176,4 | 177,8 | 176,6 | 176 | 176 |
| 2' | 36,5 | 36 | 34,7 | 34,3 | 41,2 | 41,2 | 41 |
| 3' | 18,7 | 18,5 | 19,2 | 18,5 | 26,8 | 27,1 | 27,2 |
| 4' | 13,6 | 13,61 | 18,7 | 19,3 | 11,7 | 11,7 | 11,8 |
| 5' |  |  |  |  | 16,6 | 16,7 | 16,6 |
| 18-OAc |  |  |  |  |  |  |  |
| C=O | 169,8 | 170,6 | 170,1 | 170,6 | 170,1 | 170,1 | 170,6 |
| Me | 21,2 | 21,9 | 21,2 | 21,3 | 21,2 | 21,5 | 21,3 |
| 19-OAc |  |  |  |  |  |  |  |
| C=O | 170,1 | 170,7 | 169,7 | 170,7 | 169,8 | 169,8 | 170,7 |
| Me | 21,4 | 21,4 | 21,4 | 21,7 | 21,7 | 21,3 | 21,4 |

* in the publication of Chen et al (2008), 17-C and 20-C are interchanged compared to other publications cited for the structural determination of corymbulosin I, caseamembrin E and isocaseamembrin E.

Chart 1: ^1^H and ^13^C assignments of compounds **1**-**3**: caseamembrin T (**1**), corymbulosine I (**2**), and isocaseamembrin E (**3**), respectively.

Figure 1S: HPLC chromatogram of fraction B containing compounds **1**-**3**, eluted at 34.4 min, 35.5 min and 38.7 min, respectively.

Figure 2S : H^1^ NMR spectra of **1**.

Figure 3S : H^1^ NMR spectra of **2**.

Figure 4S : H^1^ NMR spectra of **3**.
